# Chemical Composition, Toxicity, Antinociceptive, and Anti-Inflammatory Activity of Dry Aqueous Extract of *Varronia multispicata* (Cham.) Borhidi (*Cordiaceae*) Leaves

**DOI:** 10.3389/fphar.2019.01376

**Published:** 2019-11-27

**Authors:** Klaylton Lopes, Juliana Oliveira, Fabio J. C. Sousa-Junior, Túlio da F. Santos, Débora Andrade, Sara L. Andrade, Washington L. Pereira, Paulo Wender P. Gomes, Marta C. Monteiro, Consuelo Y. Yoshioka e Silva, Milton Nascimento da Silva, Cristiane F. Maia, Enéas A. Fontes-Júnior

**Affiliations:** ^1^Programa de Pós-Graduação em Ciências Farmacêuticas, Instituto de Ciências da Saúde, Universidade Federal do Pará, Belém, Brazil; ^2^Laboratório de Farmacologia da Inflamação e do Comportamento, Universidade Federal do Pará, Belém, Brazil; ^3^Laboratório de Cromatografia Líquida, Instituto de Ciências Exatas e Naturais, Universidade Federal do Pará, Belém, Brazil; ^4^Programa de Pós-Graduação em Saúde e Produção Animal na Amazônia, Universidade Federal Rural da Amazônia, Belém, Brazil

**Keywords:** *Varronia multispicata* (Cham.) Borhidi, antinociceptive, anti-inflammatory, toxicity, folk medicine, flavonoids

## Abstract

*Varronia multispicata* (Cham.) Borhidi (*Cordiaceae*), an herbaceous plant distributed in tropical and subtropical regions is native of Brazil and widely used in folk medicine to treat respiratory and digestive diseases, inflammation, and some types of infections. Thus, this study aimed to investigate acute oral toxicity, antinociceptive, and anti-inflammatory activities of dry aqueous extract of *V*. *multispicata* (AEVm) and to identify its compounds. Extract was obtained by lyophilized leaf infusion and its composition was analyzed by ultra-performance liquid chromatography-high resolution mass spectrometry (LC-MS). Acute oral toxicity was evaluated in female rats treated with AEVm (2,000 mg/kg) in a single oral dose. Mortality, body weight changes, feed and water intake, organ weights, histological and biochemical parameters were screened for 14 days. Antinociceptive activity was evaluated by writhing (WT), formalin (FT), and hot plate (HP) tests in male mice while anti-inflammatory activity was performed by carrageenan (CPE) and dextran (DPE)-induced paw edema tests and carrageenan-induced peritonitis (CP) test in male rats. Additionally, spontaneous open-field (OF) locomotion was evaluated. LC-MS analysis revealed the presence of flavonoids with biological activity. In toxicity evaluation, extract did not cause deaths in dose of 2,000 mg/kg, and there were no significant behavioral or biochemical alterations. Additionally, evidence of hepatoprotective and antioxidant activity was observed. In pharmacological evaluation AEVm showed dose-dependent antinociceptive activity in WT, with a median effective dose of 146.89 mg/kg, which showed selectivity by inflammatory base processes (FT first phase; p < 0.001), showing no activity in neuropathic nociception components (FT second phase and HP) or about consciousness and locomotion in OF. AEVm also showed significant anti-inflammatory activity, inhibiting CPE (p < 0.001) and cell migration (p < 0.05) and nitric oxide (NO) production (p < 0,01) in CP test. These data demonstrate that AEVm has low oral toxicity—with evidence of hepatoprotective and antioxidant properties—antinociceptive and anti-inflammatory activity, supporting *V. multispicata* traditional use, possibly related to flavonoids present in its constitution.

## Introduction

Inflammation and pain are clinical conditions present in most pathologies, being among main sources of dysfunctional and disabling conditions, requiring pharmacological intervention (Nathan and Ding, 2010; Henschke et al., 2015). Currently the most commonly used medications are steroidal and non-steroidal anti-inflammatory drugs (NSAIDs), and central-acting analgesics (Conforti et al., 2009; Kunanusorn et al., 2009).

In fact, NSAIDs are among most widely used medications due to their efficacy for a wide range of pain and inflammatory conditions. However, their long-term administration may induce several adverse effects such gastro-intestinal ulcers, hepatotoxicity, bleeding, renal disorders, and immunosuppression (Henzen, 2003; Wirtha et al., 2006). Opiates are most effective in cases of moderate to severe pain, although requires the clinical management of risks associated with side effects, abuse and dependence (Rosenblum et al., 2008). Therefore, development of more powerful and safe anti-inflammatory and analgesic drugs is still needed as alternatives to these drugs limitations (Dharmasiri et al., 2003; Kumara, 2001).

Plants inserted in traditional medicine have interested scientific community as a source of new bioactive substances discovery for human disorders treatment (Novais et al., 2003; Samy et al., 2008). In this sense, plants with therapeutic potential are a promising strategy for the development of anti-inflammatory drugs in search of a better therapy, reinforcing the importance of ethnopharmacological knowledge (Gupta et al., 2006; Phanse et al., 2012; Hegde et al., 2014).

The *Varronia* P. Browne genus was originally attributed to *Boraginaceae* as a subgenus of *Cordia* L. (Schlechtendal and Chamisso, 1830; Gottschling et al., 2005; Gurke, 1893; Miller and Wood, 2008), however, morphological and molecular studies have indicated *Varronia* as a sister-like of *Cordia*, thus recognizing it as a distinct genus (Gottschling et al., 2005; Miller and Gottschling, 2007). In addition, phylogenetic evidence defined *Boraginaceae* as non-monophyletic, altering the classification between subfamilies and family, as well as their intrinsic species (Gottschling et al., 2005; Miller and Gottschling, 2007). Even after segregation, *Varronia* remains one of the largest genera of *Cordiaceae* (*Boraginales*), comprising about 130 neotropical species distributed from Mexico to central regions of Southern America (Miller and Gottschling, 2007). In Brazil, there are about 30 of *Varronia* species widely distributed in Amazon Forest, Atlantic Forest, Cerrado, and Caatinga vegetation (Stapf, 2015).

*Varronia multispicata* (Cham.) Borhidi (family: *Boraginaceae* Juss; Genus: *Varronia* P. Browne), synonyms *Cordia multispicata* Cham. (basionym), *Cordia bahiensis* DC., and *Lithocardium multispicatum* (Cham.) Kuntze, is an herbaceous plant, native of Brazil and mainly distributed in the Amazon area (Rapisarda et al., 1997; Vieira and Silva, 1997; Kuroyanagi et al., 2001; Stapf, 2015; Forzza et al., 2016). This plant is locally known as “maria-preta,” “carucaá,” and “*Cordia*” (Zoghbi et al., 2010). In folk medicine, tea is widely used as an expectorant, for digestive diseases, as a drug for contusion and for some types of infection (Kuroyanagi et al., 2001). Pharmacological and phytochemical studies have reported several ursane-, oleanane-, and dammarane-type triterpenes isolated as potential antiandrogen constituents in the ethyl acetate fraction of the methanolic extract from the leaves of this species (Kuroyanagi et al., 2001; Kuroyanagi et al., 2003). In addition, sesquiterpenes were the predominant constituent class in the oils of *V. multispicata* and other *Varronia* syn. *Cordia* species (Santos et al., 2006; Oliveira et al., 2007; Diniz et al., 2008; Zoghbi et al., 2010; Rodrigues et al., 2012).

Several studies have shown in other species of *Varronia* a diversity of constituents and properties, revealing ethnopharmacological and chemotaxonomic importance of this genus. Phytochemical reports indicated presence of monoterpenes (Fun et al., 1990car), triterpenes (Kuroyanagi et al., 2001; Kuroyanagi et al., 2003), sesquiterpenes (Zoghbi et al., 2010; Rodrigues et al., 2012), tannins, and flavonoids (Hormaza et al., 2014). In addition, investigations led to the isolation of larvicidal and antifungal meroterpenoid naphthoquinones from roots of *Varronia linnaei* (Stearn) J.S. Mill. (syn. *Cordia linnaei* Stearn) and *Varronia curassavica* Jacq. (syn. *Cordia curassavica* (Jacq.) Roem. & Schult. or *Cordia verbenacea* DC.) (Ioset et al., 1998; Ioset et al., 2000), cytotoxic meroterpenoid benzoquinones against several cancer cell lines from *Varronia globosa* Jacq. [syn. *Cordia globosa* (Jacq.) Kunth] (Menezes et al., 2005), and anti-inflammatory sesquiterpenes isolated from the essential oil of *Varronia curassavica*, that became the basis of a phytotherapic drug (Passos et al., 2007; Dutra et al., 2016).

Based on its use in traditional medicine, present study aimed to investigate chemical composition and effects of dry aqueous extract of leaves of *V. multispicata* in standard models of toxicity, nociception, and inflammation in rodents.

## Materials and Methods

### Chemicals and Reagents

Chromatographic-grade acetonitrile was supplied by Tedia (Fairfield, OH, USA) and formic acid from Thermo Fisher Scientific Inc. (Waltham, MA, USA). Ultrapure water obtained by a Direct-Q 5 system from Millipore (Merck, Darmstadt, Germany). TBA, DTNB, ABTS, and Trolox were obtained from Sigma-Aldrich (Darmstadt, Germany) and ALT, AST, GGT, and ALP activity assay kits were obtained from VIDA Biotecnologia (MG, Brazil). All chemicals used were of analytical grade. Acetic acid and formaldehyde (Vetec Química Fina, RJ, Brazil); indomethacin, carrageenan, dextran, cyproheptadine hydrochloride (Sigma-Aldrich, MO, USA); morphine sulfate, and naloxone hydrochloride (Cristália, RJ, Brazil).

### Plant Collection, Identification, and Preparation of Dry Aqueous Extract

*V. multispicata* (Cham.) Borhidi leaves were collected at São Francisco of Pará, Pará state, Brazil (1°18’27.3”S, 47°45’15.0”W), in January 2013 at 24°C and 85% relative humidity. The botanical identification was performed by the specialist Dr. Silvane Tavares Rodrigues from Brazilian Agricultural Research Corporation (Embrapa) Eastern Amazon (Pará-Brazil) and a voucher specimen was deposited in its IAN Herbarium, under the code 188979.

Plant leaves were washed with tap water and 0.1% aqueous NaOCl (sodium hypochlorite) solution and dried in forced-air drying oven at 45°C until constant weight. Dried leaves were crushed using a knife mil obtaining 200 g of moderately fine powder (particle size 355 µm). Powder was infused in 2 L of ultrapure water at 100°C, remaining immersed for 24 h. The solution was filtered, immediately frozen, and then lyophilized (ALPHA 2-4 LDplus) at −20°C. Freeze-drying process yielded 18 g of dry aqueous extract of *V. multispicata* (AEVm) which were kept in a vacuum desiccator.

### Ultraperformance Liquid Chromatography Coupled With Electrospray Ionization Tandem Quadrupole Time-Of-Flight Mass Spectrometry Analysis

LC-MS analysis was performed on an ultraperformance liquid chromatography (UPLC) system coupled to a Xevo G2-S QTof mass spectrometer equipped with an electrospray ionization (ESI) source (Waters, Milford, MA, USA). Separation was performed on a BEH C18 column (Waters, Wexford, Ireland; 50 × 2.1 mm i.d., 1.7 µm particle size) at 40°C with a gradient elution programmed at constant flow rate (0.3 ml.min^−1^). The extract was analyzed at concentration of 1 mg.ml^−1^ injected (5 µl) simultaneously with leucine-enkephalin (reference compound). Mobile phase consisted of 0.1% formic acid aqueous solution (A) and 0.1% formic acid in acetonitrile (B). A linear gradient was performed ranging from 5 to 95% B in 10 min. ESI source was operated in positive mode over a wide mass range (m/z 50–1,200) with a scan time of 0.1 s. The source temperature was set at 150°C with a cone gas flow of 20 L h^−1^. Desolvation gas flow was set at 600 L.h^−1^ at a temperature of 450°C. Capillary was set at 3.0 kV with cone voltage at 20 V. MassLynx Software (Waters, Milford, MA, USA) was used for data acquisition and processing.

### Animals

Two-month-old male Swiss albino mice (25–30 g) and male and female Wistar rats (150–200 g) were obtained from Central Housing Facility of Evandro Chagas Institute (IEC). Animals were kept in collective cages (five animals per cage) under standard conditions of temperature (22 ± 1°C), humidity (50–60%), light/dark cycle (12 h) and feed (standard pellet diet), with water *ad libitum*. They were acclimated to laboratory for 12 h before the experiments, only with available water at will. All protocols were conducted according to the Guide for the Care and Use of Laboratory Animals (2011) and approved by the Animal Use Ethics Committee of the Federal University of Pará (CEUA/UFPA; license numbers 62-2015 and 6029300817).

### Drug Treatment

AEVm, standard drugs, and phlogistic or nociceptive agents were dissolved in 0.9% saline, except for acetic acid, which was solubilized in distilled water. Saline was therefore adopted as control. All treatments were performed with a standard volume of 0.1 ml/10 g body weight for mice and 0.1 ml/100 g body weight to rat by oral (gavage; po), subcutaneous (sc), or intraperitoneal (ip) way.

### Acute Oral Toxicity

#### Treatment and Hippocratic Screening

Acute oral toxicity was evaluated in female Wistar rats (n = 6/group) according to the Guidelines for Testing Chemicals n° 420 of the Organization for Economic Cooperation and Development (OECD, 2001). After acclimatization procedure, animals were treated with saline (control) or the limit dose (2,000 mg/kg) of AEVm. Then animals were evaluated for signs of toxicity, according to parameters related by Malone (1977), every hour for the first 4 h and daily thereafter for 14 days. Feed and water intake and weight gain were also verified daily, as well as the incidence of deaths.

On 14^th^ day the surviving animals were anesthetized and euthanized by cervical dislocation to collect organ (stomach, liver, kidneys and lungs), destined for macroscopic and histopathological evaluation, and blood samples, for biochemical evaluations.

#### Biochemical Assays

##### Sample

Blood was collected by ventricular puncture in tubes containing ethylenediaminetetraacetic acid (EDTA). Plasma and erythrocyte fractions were separated by centrifugation for 10 min at 1,400 ×g. Uppermost erythrocytes were discarded and the remaining washed three times in 0.9% saline until a clean suspension (2 ml) of erythrocyte pellet at approximately 50% hematocrit. Washed erythrocytes were diluted 1:10 with saline and 500 µl then lysed by adding 2,500 µl distilled water (hemolysate). From plasma were analyzed the alanine aminotransferase (ALT), aspartate aminotransferase (AST), gamma glutamyl transpeptidase (γ-GT), and alkaline phosphatase (ALP) activities. Trolox equivalent antioxidant capacity (TEAC), lipid peroxidation (through malondialdehyde levels; MDA), and nitrite concentration were also analyzed in plasma. Reduced glutathione (GSH) level was measured in hemolysate.

##### Oxidative Biochemistry Assays

*Total Antioxidant Capacity*. It was evaluated by TEAC assay, according to method of Miller et al. (1993), adapted by Re et al. (1999), which measures the ability of antioxidants present in sample to scavenge the stable ABTS·^+^ (2,2′-azinobis, 3-ethylbenzothiazoline-6-sulfonic acid) cation radical, a blue-green chromophore. Antioxidants cause a reduction in absorption at a wavelength of 734 nm proportional to their potency and concentration. The antioxidant capacity of AEVm was then measured after 4 min from addition of 10 µl plasma in 1 ml ABTS·^+^. Results expressed as Trolox equivalent (mmol/L) *via* calibration curve (r^2^ = 0.999).

*Reduced Glutathione Level*. According to Ellman (1959) and Schalcher et al. (2014) GSH level can be determined based on its ability to reduce dithiobis-2-nitrobenzoic acid (DTNB) to nitrobenzoic acid (TNB). Therefore, 20 µl of the hemolysate were solubilized with distilled water plus PBS/EDTA (3 ml) and immediately spectrophotometrically read at 412 nm. Then 100 µl of DTNB were added and read again after 3 min. Results were expressed in µmol/ml.

*Nitric Oxide Concentration*. Plasma nitric oxide (NO) concentration was measured indirectly by Griess methods (Granger et al., 1999). Firstly, the sample nitrate was converted to nitrite with nitrate reductase. Then 100 µl of plasma were incubated with equal volume of Griess reagent for 10 min at room temperature and after read on a spectrophotometer at 570 nm. Nitrite concentrations were calculated based on sodium standard nitrite (NaNO_2_) curve and results expressed in µmol/L.

*Lipid Peroxidation Level*. Lipid peroxidation level was evaluated based on the reaction of its product, polyunsaturated fatty acid metabolite, MDA with thiobarbituric acid (TBA) (Liu et al., 2014a; Yang et al., 2015). For this, 1 ml of TBA solution was mixed with 500 µl of sample. The resulting solution was read on a spectrophotometer at 535 nm. Concentrations were calculated based on the standard MDA curve and the results expressed in nmol/ml.

##### Hepatic Function Assays

*Alanine Aminotransferase Activity*. ALT Activity Kinetic Assay Kit has been used to determine spectrophotometrically the activity of ALT in samples. It is based on the quantification of pyruvate produced by ALT. Pyruvate and NADH are converted to lactate and NAD^+^ by the enzyme lactate dehydrogenase (LDH). The decrease in NADH absorbance at 340 nm is proportional to ALT activity expressed as U/ml (Hamada and Ohkura, 1976; Phillip and Graham, 1995).

*Aspartate Aminotransferase Activity*. AST Activity Kinetic Assay Kit (VIDA Biotechnology) has been used to determine spectrophotometrically the activity of AST in samples. AST activity assay is based on the quantification of oxaloacetate produced by AST. Oxaloacetate and NADH are converted to malate and NAD^+^ by the enzyme malate dehydrogenase (MDH). The decrease in NADH absorbance at 340 nm is proportional to AST activity expressed as U/ml (Yagi et al., 1979; Phillip and Graham, 1995).

*Gamma Glutamyl Transpeptidase Activity γ-GT* Activity Kinetic Assay Kit (VIDA Biotechnology) has been used to determine the activity of γ-glutamyl transpeptidase (*γ-GT*) in samples. It is based on *γ-GT*-mediated transfer of glutamyl groups of L-α-glutamyl-3-carboxy-4-nitroanilide to glycylglycine, yielding 5-amino-2-nitrobenzoate exhibiting a red colored product (maximal absorbance at 405 nm). The rate of the reaction is directly proportional to the enzyme activity expressed as U/ml (Szasz, 1969; Phillip and Graham, 1995).

*Alkaline Phosphatase Activity*. ALP Activity Kinetic Assay Kit (VIDA Biotechnology) has been used to determine the activity of ALP in samples. It is based on ALP-mediated hydrolysis of phosphate esters in an alkaline buffer, resulting in the formation of an organic radical and inorganic phosphate. The improved method utilizes p-nitrophenyl phosphate (PNPP) that is hydrolyzed by ALP to nitrophenol into a yellow colored product (maximal absorbance at 405 nm). The rate of the reaction is directly proportional to the enzyme activity expressed as U/ml (Szasz, 1969; Phillip and Graham, 1995).

#### Histopathological Analysis

After removed, the kidneys, liver, stomach, and lungs were weighed and then fixed in 10% buffered formalin, embedded in paraffin, sectioned (5 µm thick), processed using an alcohol-xylene series, and stained with hematoxylin and eosin (H&E). All sections were surveyed on an optical microscope (Nikon Eclipse E200).

### Antinociceptive Activity

#### Acetic Acid-Induced Writhing

According to the model proposed by Koster et al. (1959), acetic acid (0.6% v/v) was administered ip in male mice, generating nociception manifested by the occurrence of writhing. Animals (n = 6/group) were treated orally 1 h before noxious induction with 0.9% saline (control), indomethacin 10 mg/kg (standard drug), or AEVm (25, 75, 200, or 400 mg/kg). The number of writhes was verified within 10 to 30 min after acetic acid injection.

Median effective dose (ED50) for antinociceptive activity was determined by linear regression of log-dose *versus* percentage of nociception inhibition, being applied to remaining biological activity assays to reduce number of animals used.

#### Formalin Test

According to Hunskaar et al. (1985), biphasic nociception was induced by sc injection into plantar region (right hind paw) of 20 µl of formalin solution (0.92% formaldehyde). First (neurogenic) phase triggers within 5 min after formalin injection, followed by a transitional period. Second (inflammatory) phase occurs within 15 to 30 min after noxious stimulation. In both phases, nociception is evidenced by licking the injected paw.

For antinociceptive evaluation, male mice (n = 6/group) were pretreated (1 h) orally with 0.9% saline (control) or AEVm (ED50 to WT). Standard drug (morphine 4 mg/kg) was administered by sc way 30 min earlier noxious induction. Time of nociception manifestation was recorded (in seconds) in both phases of test.

To verify opioid system involvement in antinociceptive activity, three other groups received naloxone (0.4 mg/kg), a nonselective opioid antagonist, by sc way 15 min before the treatments described above.

#### Hot Plate Test

AEVm effect on supraspinatus nociception processes was evaluated by MacDonald *et al*. (1946) method, which consists of exposing mice to a plate (Ugo Basile, model 35100, Varese, Italy) heated to 50 ± 0.5°C. Nociception is manifested by licking the hind paws, jumping, or shaking. One day before the experiment, mice with response latency up to 20 s were selected.

In experiment, male mice (n = 6/group) were treated orally with 0.9% saline (control) or AEVm (ED50 to WT). Standard drug (morphine 4 mg/kg) was administered by sc way. After 60 min of treatment (30 min for morphine), latency for nociception manifestation was recorded (in seconds) at 0, 30, 60, 90, and 120 min. Cut-off time of 30 s was fixed to avoid damage to the paws.

#### Open Field Test

Mice submitted to FT and HP test, except for morphine-treated groups, were subjected to open field testing 5 min prior to exposure to noxious stimuli to verify possible impairment of consciousness or mobility. Animals were exposed for 5 min on two successive days prior to the test for habituation (Rauf et al., 2017).

In the test, was followed the protocol adapted from Shahed-Al-Mahmud and Lina (2017), positioning the animals (n = 10/group) individually in the center of the arena (100 × 100 × 40 cm) and their spontaneous locomotion recorded for 5 min by a camcorder. Videos were analyzed using ANY-maze™ software (Stoelting, USA), determining the total distance traveled. All tests were run between 12:00 AM and 5:00 PM in a sound-attenuated room under low-intensity light (12 lux).

### Anti-Inflammatory Activity

#### Paw Edema Test

To evaluate anti-inflammatory properties, edema was induced in the rats right hind paw by sc injection of 100 µl of carrageenan (1%, w/v saline; Winter et al., 1962) or dextran (1%, w/v saline; Carvalho et al., 1999). The left hind paw was used as volume control, receiving sc injection of 100 µl 0.9% saline. Animals (n = 6/group) were treated orally 1 h before edema induction with 0.9% saline (control) or AEVm (ED50 to WT). Standard groups received indomethacin 10 mg/kg (carrageenan-induced model) or cyproheptadine 10 mg/kg (dextran-induced model). Evolution of edema, that was defined as the volume (ml) difference between the right and left hind paws, was measured using digital plethysmometer (Ugo Basile, model 7140, Varese, Italy) at 0, 1, 2, 3, 4, and 5 h after carrageenan injection or at 0, 30, 60, 90, and 120 min after dextran injection.

#### Carrageenan-Induced Peritonitis

According to proposed by Souza and Ferreira (1985), cavitary inflammation was induced in rats (n = 6/group) by ip injection of carrageenan (0.3 mg/kg) 1 h after oral treatment with saline (0.9%), AEVm (146.89 mg/kg), or dexamethasone (1 mg/kg). Four hours after phlogistic induction the animals were euthanized, and 10 ml of PBS were injected into peritoneal cavity. Subsequently, peritoneal wash was collected to evaluate NO production and leukocyte migration.

NO production was indirectly evaluated as described above and nitrite concentration was corrected as a function of protein concentration (Bradford, 1976) in sample. Results were expressed as nmol/mg.

##### Cell Migration Assessment

For total leukocyte count, 80 µl of peritoneal wash were diluted in 320 µl of Turk’s solution and resulting 400 µl were transferred to Neubauer chamber, counting by optical microscopy (400x magnification). Results were expressed as number of cells x 10^7^/ml.

### Statistical Analysis

All data were processed by SigmaPlot 14.0 software and expressed as mean ± standard error of the mean (SEM). Distribution was evaluated by Kolmogorov-Smirnov test. Difference between groups was evaluated by Student’s t-test and one-way ANOVA, with or without repeated measurements (RM), followed by Holm Sidak *post hoc* test. Differences of P < 0.05 were considered as statistically significant.

## Results

### Liquid Chromatography-High Resolution Mass Spectrometry Analysis of Aqueous Extract of *Varronia multispicata*

**Figure 1:**
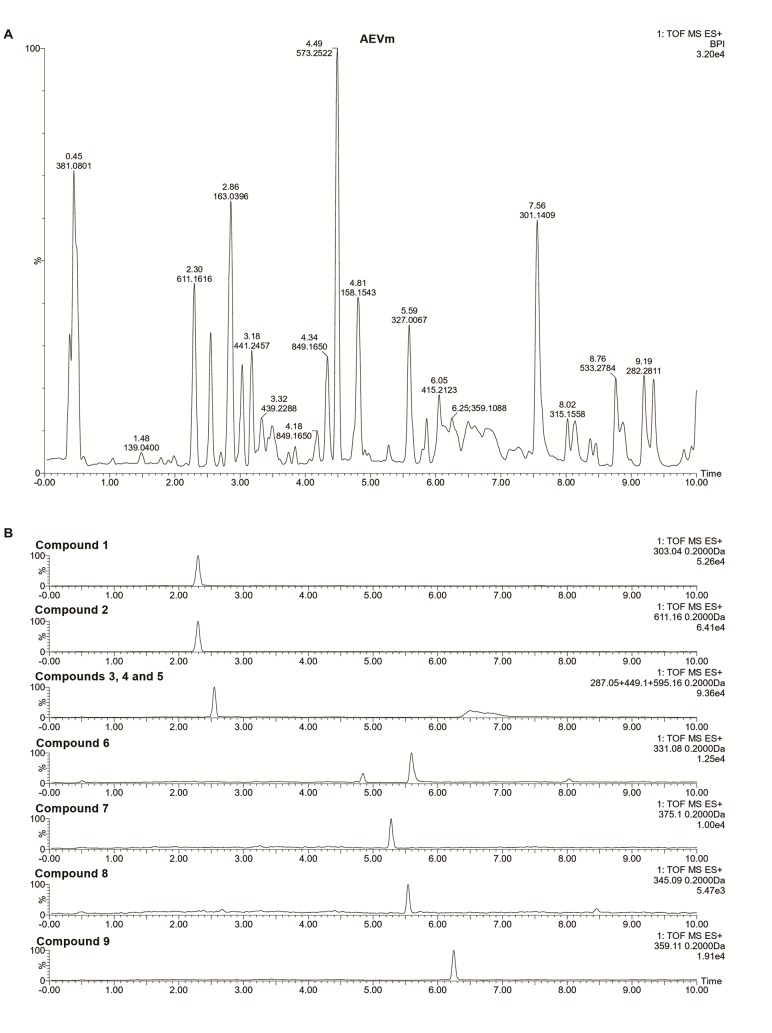
Total ion current chromatograms of aqueous extract of Varronia multispicata **(A)** and representative chromatograms of isolated flavonoids **(B)**.

The liquid chromatography coupled to mass spectrometry (UPLC-ESI-HRMS) analysis of the sample of AEVm used in the present investigation allowed the identification of nine constituents. The total ion chromatogram of the AEVm (**Figure 1A**) illustrated the m/z ratio of constituents of the extract and their corresponding retention time. Most of the substances identified belongs to flavonoids groups, which are shown in the **Table 1**. Among these constituents, nine flavonoids were isolated by our group, and their mass spectra are illustrated in **Figure 1B**.

**Table 1:** Flavonoids identified by liquid chromatography-high-resolution-electrospray ionization-mass spectrometry in aqueous extract of *Varronia multispicata*.

Peak	RT (min)	Compound	Formula	[M+H]^+^		
				*m/z* Theo	*m/z* Exp	Error (ppm)
**1**	2.3	Quercetin	C_15_H_10_O_7_	303.0505	303.0498	2.30
**2**	2.3	Quercetin 3-O-robinobioside	C_27_H_30_O_16_	611.1612	611.1616	0.65
**3**	2.5	Kaempferol	C_15_H_10_O_6_	287.0556	287.0567	3.83
**4**	2.5	Kaempferol 7-O-glucoside	C_21_H_20_O_11_	449.1084	449.1077	1.55
**5**	2.5	Kaempferol 3-O-rutinoside	C_27_H_30_O_15_	595.1663	595.1644	3.20
**6**	4.8	3,7-Dimethoxy-5,3’, 4’-trihydroxyflavone	C_17_H_14_O_7_	331.0818	331.0838	6.04
**7**	5.2	5,6’-Dihydroxy-7,2’,4’,5’-tetramethoxyflavone	C_19_H_18_O_8_	375.1080	375.1071	2.40
**8**	5.5	5,3’-Dihydroxy-3,7,4’-trimethoxyflavone	C_18_H_16_O_7_	345.0974	345.0983	2.60
**9**	6.2	5-Hydroxy-3,7,3’,4’-tetramethoxyflavone	C_19_H_18_O_7_	359.1131	359.1126	1.40

### Aqueous Extract of *Varronia multispicata* Acute Oral Toxicity

#### Aqueous Extract of *Varronia multispicata* Does Not Cause Deaths or Impair General Behavior, Physical, or Histological Aspects

No manifestations of toxicity were observed immediately after acute oral administration of AEVm 2,000 mg/kg and for following 14 days, with no allergic manifestations, behavioral or motor changes. Similarly, there were no deaths.

**Figure 2:**
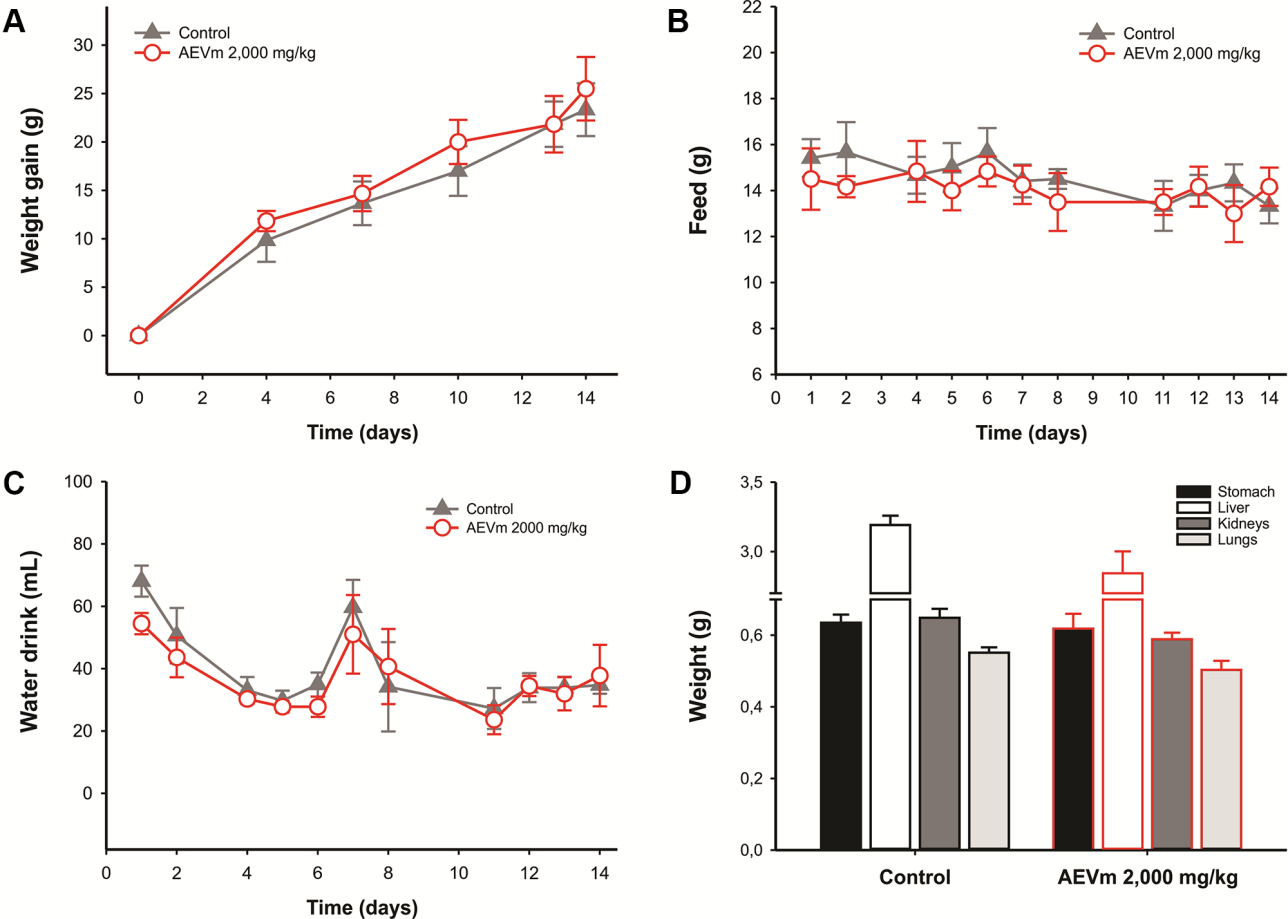
Effect of acute oral administration of aqueous extract of *Varronia multispicata* (2,000 mg/kg) on **(A)** body weight gain, **(B)** water, and **(C)** food intake over the following 14 days, and relative organ-body weight **(D)** at the end of the evaluation period. Data expressed as mean ± SEM (n = 6/group).

In addition, there were no prejudice in body weight gain and feed and water intake compared to the control group (**Figures 2A–C**), nor were there any changes in macroscopic aspects (morphology, color and size) and relative weight of stomach, liver, kidneys, and lungs (**Figure 2D**). Histopathological evaluation revealed no abnormalities in gastric, pulmonary, hepatic, and renal tissues (data not shown).

#### Aqueous Extract of *Varronia multispicata* Provides Protection Against Oxidative Damage

**Figure 3:**
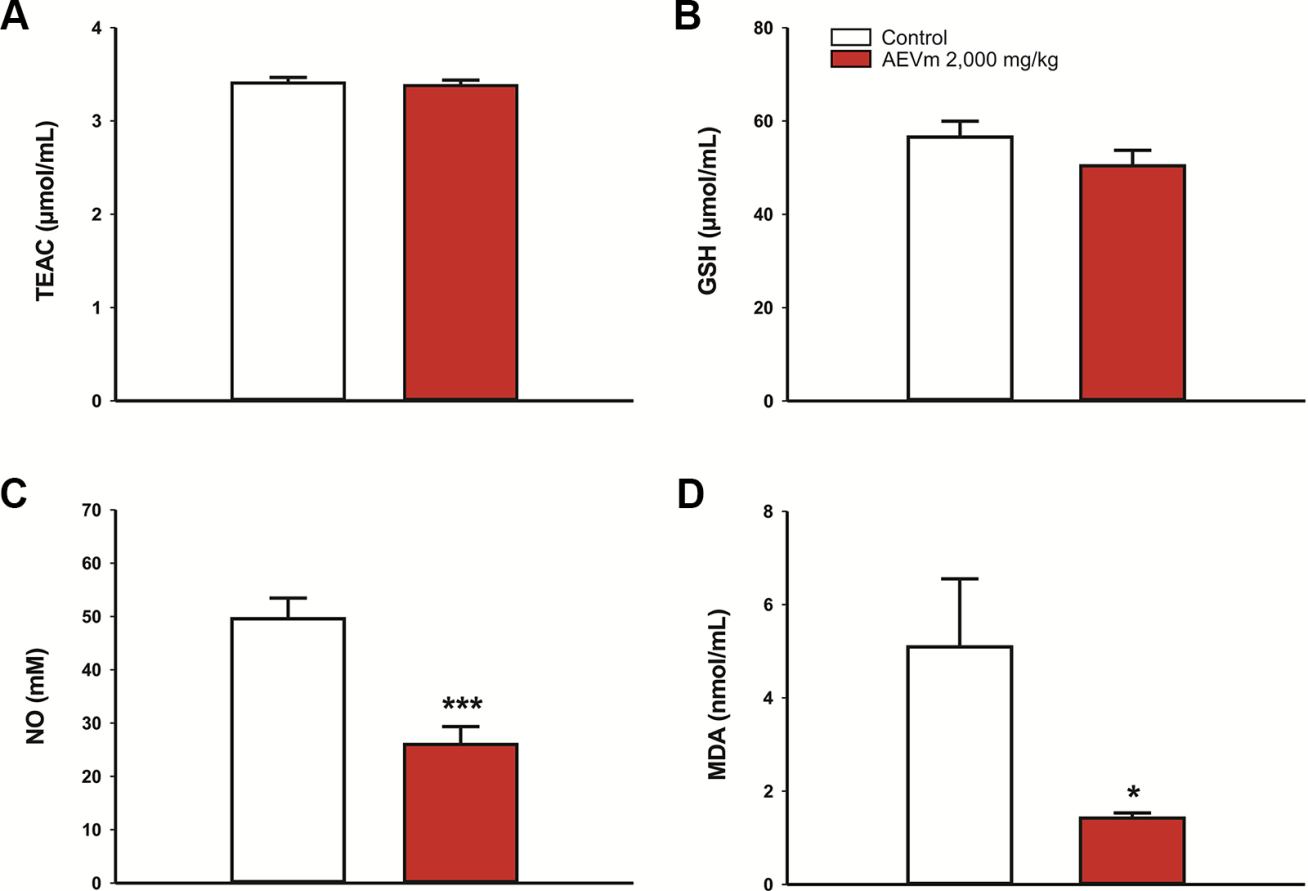
Effect of acute oral administration of aqueous extract of *Varronia multispicata* (2,000 mg/kg) on oxidative balance after 14 days of follow-up, considering **(A)** antioxidant activity and plasma levels of **(B)** GSH, **(C)** nitrite-NO, and **(D)** malondialdehyde-MDA. Data expressed as mean ± SEM (n = 6/group). ***p < 0.001; *p < 0.05 versus control. Student’s t-test.

When evaluating animal plasma samples at end of observation period, i.e., on day 14 after acute oral extract administration (2,000 mg/kg), no antioxidant activity was detected (**Figure 3A**), as well as no change in GSH levels (**Figure 3B**). Despite this, the extract significantly reduced nitrite and MDA plasma levels (p < 0.001 and p < 0.05 respectively *versus* control; **Figures 3C, D**).

#### Aqueous Extract of *Varronia Multispicata* Does Not Interfere With or Down-Regulate Liver Enzymes

**Figure 4:**
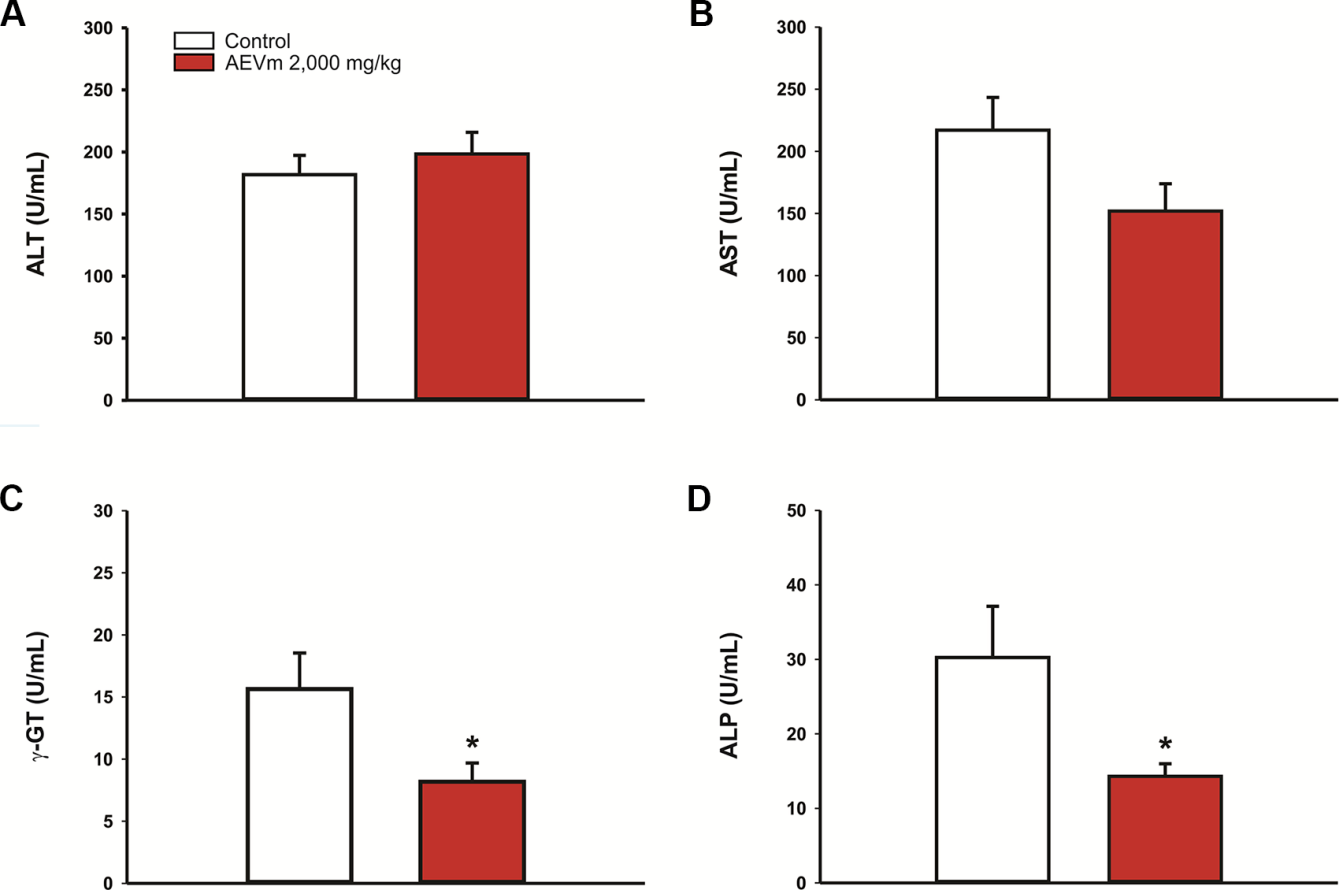
Effect of acute oral administration of aqueous extract of *Varronia multispicata* (2,000 mg/kg) on the activity of **(A)** alanine aminotransferase, ALT; **(B)** aspartate aminotransferase, AST; **(C)** gamma glutamyl transpeptidase, γ-GT; and **(D)** alkaline phosphatase, ALP. Data expressed as mean ± SEM (n = 6/group). *p < 0.05 versus control (Student’s t-test).

In the same context of previous evaluation, animals treated with AEVm maintained serum levels of ALT and AST equivalent to those found in controls (**Figures 4A, B**). On the other hand, the extract promoted significant reductions in γ-GT and ALP levels (p < 0.05 *versus* control; **Figures 4C, D**).

### Antinociceptive Activity

#### Aqueous Extract of *Varronia multispicata* Inhibits Nociception in a Dose-Dependent Pattern

**Figure 5:**
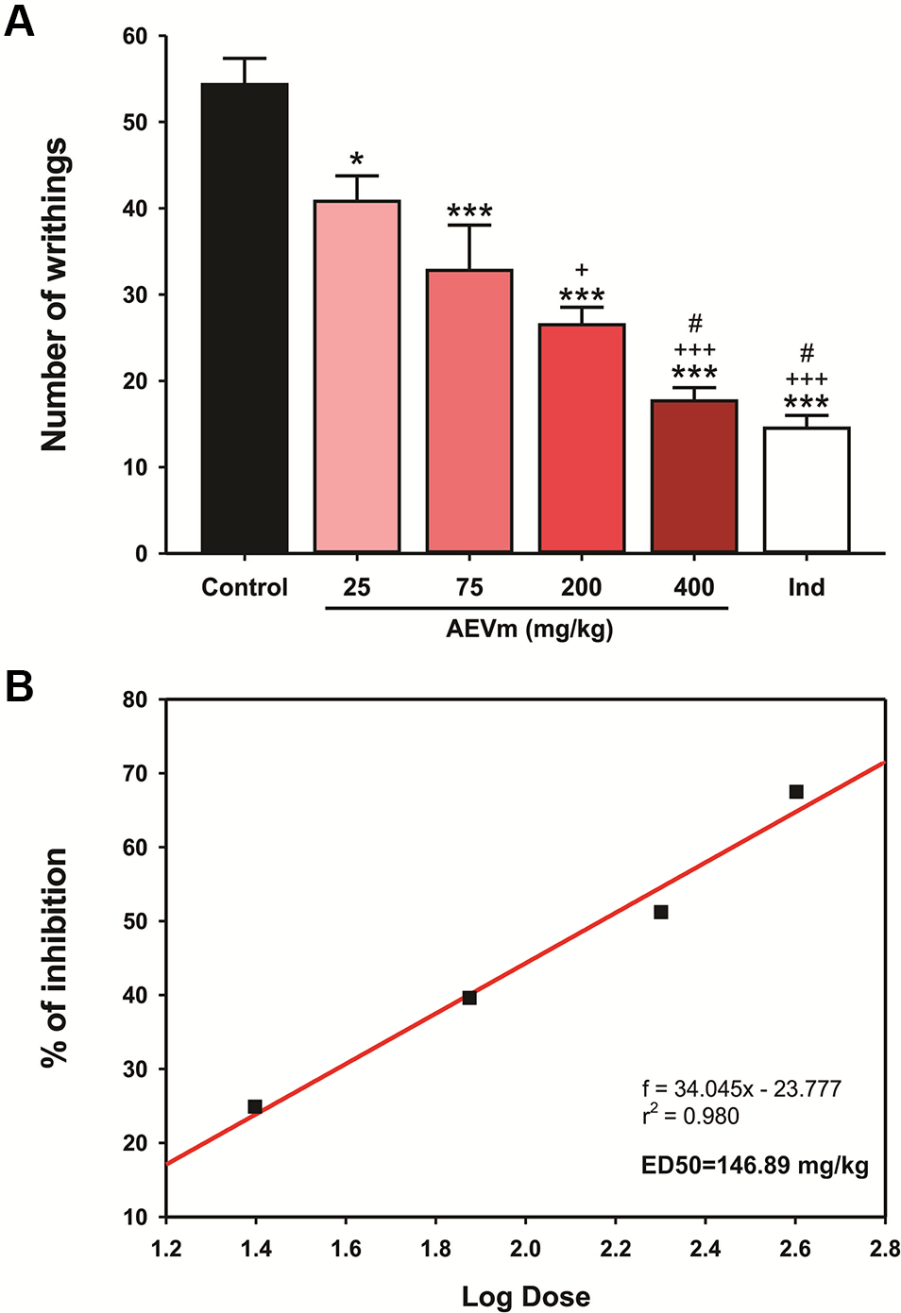
Effect of aqueous extract of *Varronia multispicata* (AEVm) (25, 75, 200, and 400 mg/kg; o.r.) on **(A)** acetic acid (0.6%)-induced abdominal writhing and **(B)** determination of median effective dose-ED50. Data expressed as mean ± SEM (n = 6/group). ***p< 0.001, *p < 0.05 versus control; *^+^* *p < 0,05,* *^+++^* *p < 0.001 versus AEVm 25 mg/kg;* *^#^* *p < 0.05 versus 75 mg/kg (ANOVA, Holm-Sidak’s test)*.

Oral pretreatment with AEVm (25, 75, 200, and 400 mg/kg) 1 h before WT induced a dose-dependent inhibition (p < 0.05, 0.001, 0.001, and 0.001 *versus* control) of acetic acid-induced abdominal writhes in mice when compared to control group (**Figure 5A**), with a median effective dose (ED50) of 146.89 mg/kg (**Figure 5B**). In addition, group treated with AEVm 400 mg/kg showed a mean reduction in nociception manifestation of 70%, reaching levels similar to animals treated with indomethacin 10 mg/kg (standard drug; 73%).

#### Aqueous Extract of *Varronia multispicata* Does Not Compromise Locomotion and Exploration in Open Field

**Figure 6:**
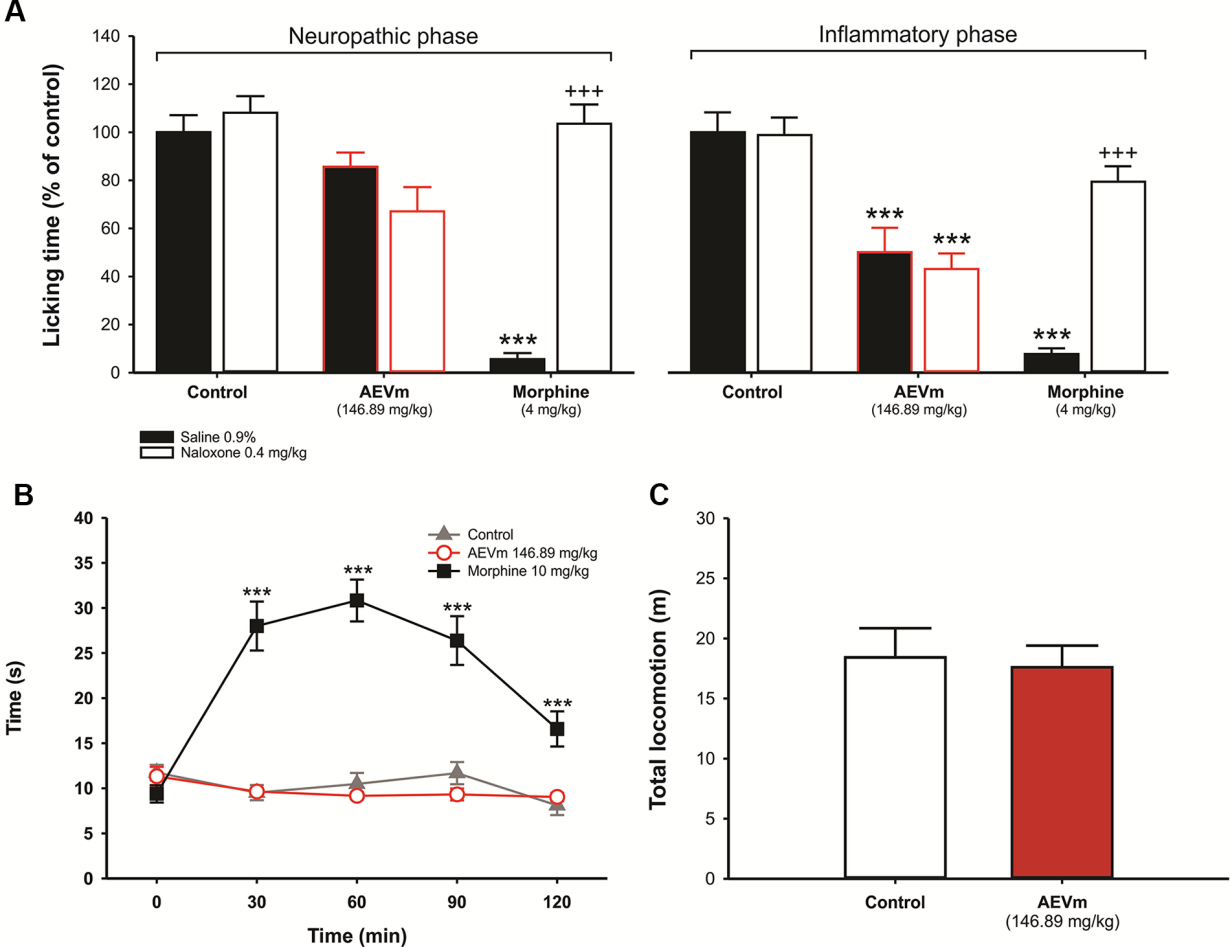
Effect of aqueous extract of *Varronia multispicata* (AEVm) (146.89 mg/kg; o.r.) on neuropathic and inflammatory nociception in formalin test with evaluation of opioid pathway participation **(A)**, thermal nociception in hot plate test **(B)**, and total locomotion in open field **(C)**. Data expressed as mean ± SEM of six animals/group (n = 10/group for open field). ***p< 0.001 versus control; **^+++^** *p < 0.001 versus morphine. Formalin test (ANOVA, Tukey´s test); hot plate (RM ANOVA, Holm-Sidak’s test).*

Applied to verify possible impairment of mobility or sedation-like effect, OF revealed that treatment with ED50 of AEVm does not compromise animals’ locomotion or exploration (**Figure 6C**), excluding the prejudice of such effects on antinociceptive evaluation.

#### Antinociceptive Effect of Aqueous Extract of *Varronia multispicata* Is Due to Modulation of Inflammatory Components of Pain

When AEVm ED50 was previously applied to animals undergoing FT, a significant reduction in paw licking time (P < 0.001 *versus* control group) was observed in second (inflammatory) phase (**Figure 6A**, at right). In first (neuropathic) phase of FT (**Figure 6A**, at left), as in HP (**Figure 6B**), treatment with AEVm did not interfere with animals’ nociception, which showed behavior equivalent to respective controls. Additionally, in FT, co-treatment with naloxone (0.4 mg/kg; s.c.) inhibited (p < 0.001) the morphine (4 mg/kg; s.c.) effect but not the extract effect (**Figure 6A**; white bars). This finding suggests that antinociceptive activity of AEVm does not involve opioid receptors activation.

### Anti-Inflammatory Activity

#### Aqueous Extract of *Varronia Multispicata* Inhibits Carrageenan-Induced But Not Dextran-Induced Paw Edema

**Figure 7:**
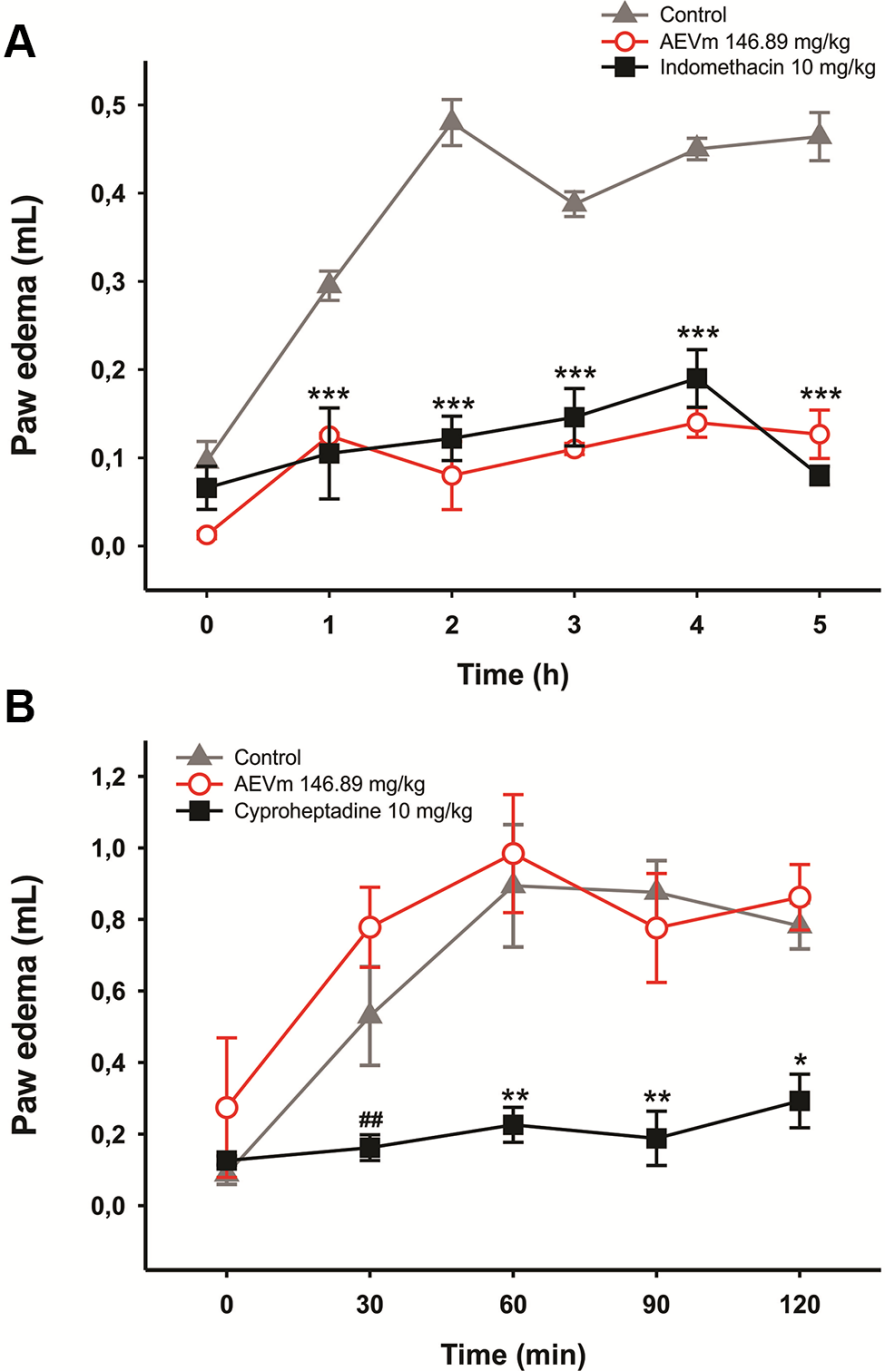
Effect of aqueous extract of *Varronia multispicata* (AEVm) (146.89 mg/kg; o.r.) on carrageenan **(A)** or dextran-induced **(B)** paw edema. Data expressed as mean ± SEM (n = 6/group). *p < 0.05, **p < 0.01, ***p < 0.001 versus control; ##p < 0.01 versus AEVm. RM ANOVA, Holm-Sidaks test.

Pretreatment with 146.89 mg/kg of AEVm significantly inhibited (p < 0.001 *versus* control) edema formation throughout the evaluation period, promoting similar effect level to that generated by standard drug (Indomethacin 10 mg/kg) (**Figure 7A**). Same was not observed for dextran-induced edema. In this test, animals treated with AEVm presented edema formation equivalent to control group (**Figure 7B**).

#### Aqueous Extract of *Varronia multispicata* Inhibits Cell Migration and Nitric Oxide Production in Peritonitis Model

**Figure 8:**
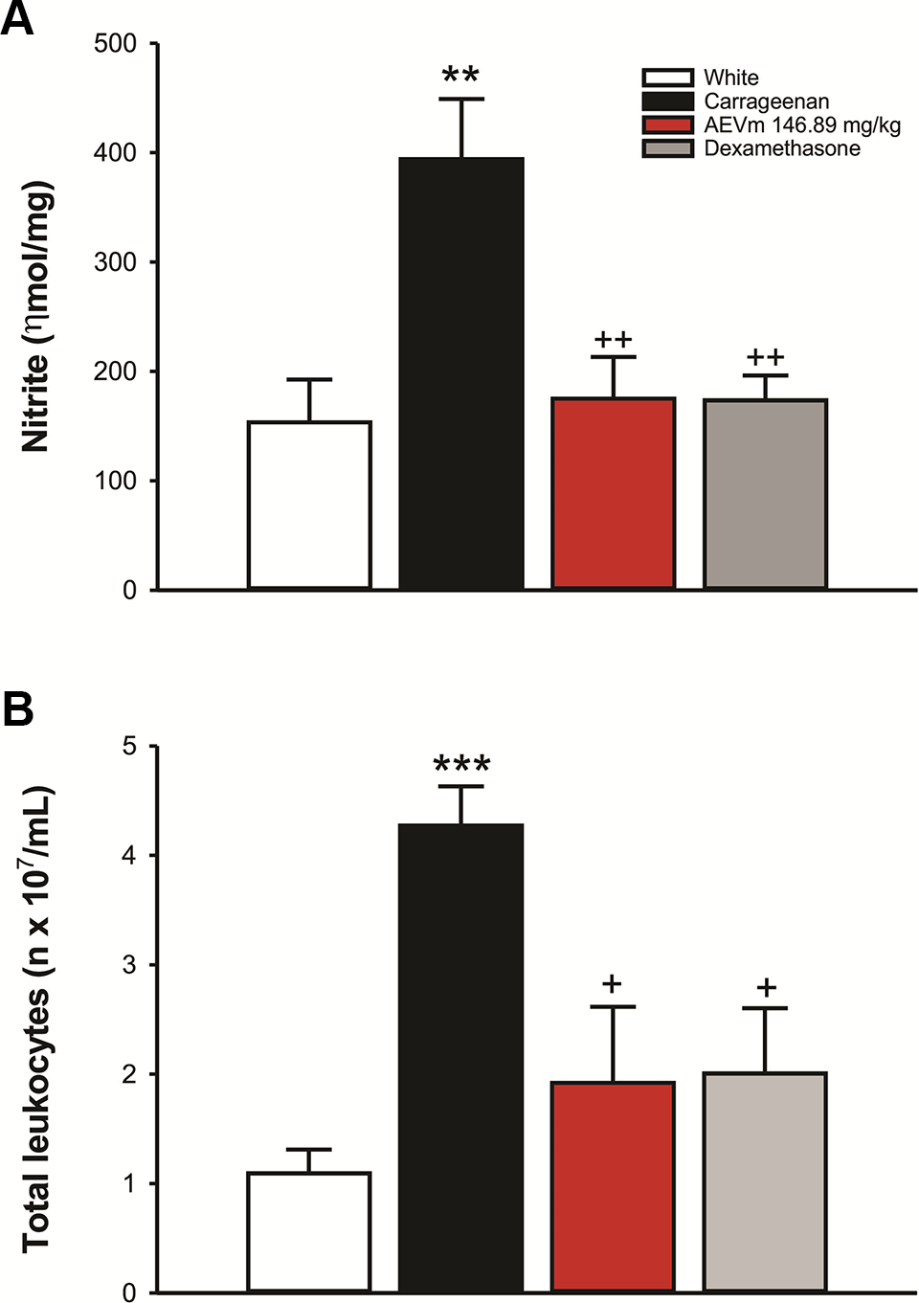
Effect of aqueous extract of *Varronia multispicata* (AEVm) (146.89 mg/kg; o.r.) on **(A)** nitrite levels and **(B)** number of leukocytes in peritoneal fluid in the carrageenan-induced peritonitis model. Data expressed as mean ± SEM (n = 6/group). **p < 0.01, ***p < 0.001 versus White; +p < 0.05, ++p < 0.01 *versus* Carrageenan. ANOVA, Holm-Sidaks test.

In peritonitis model, as intended, carrageenan significantly increased (p < 0.01) nitrite levels in peritoneal fluid compared to white group. The treatment with 146.89 mg/kg of AEVm, in turn, inhibited (p < 0.01 *versus* carrageenan) this elevation, promoting levels of nitrite equivalent to animals that did not undergo phlogistic induction (**Figure 8A**). Similarly, the extract also inhibited (p < 0.05 *versus* carrageenan) carrageenan-induced leukocyte migration. In both cases AEVm effect was like that promoted by Dexamethasone (**Figure 8B**).

## Discussion

This study is the first to evaluate the safety of acute oral administration of AEVm and its effectiveness in treating nociceptive and inflammatory processes in murine models. This initiative came from the insertion of this plant species in Amazonian medicinal culture, where it is used to treat conditions associated with inflammation and pain (Kuroyanagi et al., 2001). However, little is known about its chemical constitution, toxicity, and pharmacological properties, evidencing the relevance of elucidating its therapeutic potential. Our findings demonstrated that AEVm has low toxicity when administered acutely orally, in addition to evidence of hepatoprotection and antioxidant effect. It also demonstrated important antinociceptive and anti-inflammatory activity.

Flavonoids: quercetin (1); quercetin-3-O-rutinoside (2); kaempferol (3); kaempferol-7-O-glycoside (4); kaempferol-3-O-rutinoside (5); 3,7-dimethoxy-5,3’,4’-trihydroxyflavone (6); 5,6’-dihydroxy-7,2’,4’,5’-tetramethoxyflavone (7); 5,3’-dihydroxy-3,7,4’-trimethoxyflavone (8); and 5-hydroxy-3,7,3’,4’-tetramethoxyflavone (9) were identified based on exact masses compared to those described in the literature and compared with patterns isolated by the group (compounds 6 to 9). Flavonoids, quercetin, and kaempferol, as well as their glycosides are widespread in the plant kingdom. Nevertheless, they have a variety of biological activities such as hepatoprotective, antioxidant, and anti-inflammatory (Mohamed et al., 2010; Costa et al., 2012; Sokkar et al., 2016). Flavones are part of another subgroup of flavonoids that are widely studied because they have various pharmacological activities, suggesting that the presence of these flavonoids may contribute to the antinociceptive and anti-inflammatory properties of AEVm found in this study.

The low toxicity was evidenced by absence of deaths following the 2,000 mg/kg dose limit administration in female rats. According to the parameters proposed by OECD (2001), any substance with LD50 between 2,000 and 5,000 mg/kg, as occurred with AEVm, is extruded as a low toxicity xenobiotic. In addition, treatment of animals at limit dose did not impair the general behavior and mobility immediately after administration or over the 14 days of follow-up. Weight gain was also not altered by treatment with extract, indicating that AEVm does not cause damage to nutrition. These data are reinforced by maintenance of feed and water intake at levels equivalent to controls. In evaluation of limit dose systemic repercussions, macroscopic aspects and relative weight of the stomach, liver, kidneys, and lungs were conserved, as well as the cellular architecture and morphology of their tissues. These data are supported by the biochemical evaluation, which showed an absence of interference with ALT and AST activity. γ-GT and ALP, on the other hand, had their activity reduced among animals treated with AEVm. Hepatocellular enzymes are important markers for diagnosis of liver lesions. ALT is considered the most specific marker of liver injury, as AST may also have its levels influenced by changes in other organs, such as the heart. γ-GT is also related to renal function, oxidative stress, and chronic inflammation. Elevation of serum ALP levels is related to hepatobiliary dysfunctions and cholestatic injuries (Olatosin et al., 2014; Wang et al., 2016). Its evaluation, therefore, acts as a predictor of possible drug hepatotoxicity, as occurs with acetaminophen, whose hepatic aggression promotes a characteristic elevation of hepatocellular enzymes (Olatosin et al., 2014). In AEVm case, our biochemical results indicate the absence of tissue damage, especially to liver (Liu et al., 2014b).

The imbalance between oxidant species production and the systemic ability to neutralize them, called oxidative stress, is an important source of xenobiotic-induced cell damage. Our findings showed that AEVm does not impair plasma total antioxidant capacity, nor does it interfere with GSH content. Despite this, the extract exhibited significant evidence of antioxidant potential, as it significantly reduced levels of MDA, a product of lipid peroxidation cell damage, compared with controls. It also reduced NO plasma content, an oxidant radical, assessed indirectly through nitrite concentrations. These properties may be related to flavonoids present in the species (Mohamed et al., 2010; Costa et al., 2012; Sokkar et al., 2016). In addition, antioxidant effect of AEVm may be involved in preserving the histological and functional normality of the liver, an organ with intense oxidative metabolism (Sokkar et al., 2016). Results presented highlight not only the safety of acute oral use of the extract, but also its possible protective effects. We consider, however, the importance of further studies on extract tolerability in subchronic and chronic models.

Based on measured safety, we selected the doses to be applied in evaluation of pharmacological properties. We started at doses equivalent to 20 and 10% of the dose tested for toxicity assessment, i.e., 400 and 200 mg/kg. The other doses were adopted according to drug effect pattern. Antinociceptive and anti-inflammatory properties of AEVm, as well as the possible related mechanisms, were verified by established murine models, starting with WT, a sensitive visceral pain model for evaluation of substances with analgesic potential (Popoola et al., 2016). Acetic acid-induced nociception combines the induction of nociceptive mediators’ expression and/or release (arachidonic acid, cyclooxygenase-2, prostaglandins E2 and F2α, bradykinin, serotonin, and histamine) and non-selective cation channels activation (Hasan et al., 2014). Therefore, it is a model of inflammatory pain and is efficiently modulated by drugs capable of inhibiting prostaglandin biosynthesis, despite the diversity of mechanisms that involve it. Our results demonstrated that AEVm significantly inhibited the writhing generated by acetic acid-induced inflammation at all doses (25, 75, 200, and 400 mg/kg) tested, showing a dose-dependent pattern with an ED50 of 146.89 mg/kg. These findings allow us to infer that AEVm may exert its effect by inhibiting the synthesis/release of inflammatory mediators, especially prostaglandins.

To verify the performance of AEVm on peripheral and central components of nociception was performed the FT. For this and other tests we adopted the WT ED50 as standard dose of AEVm, aiming to reduce the number of animals used. FT is a model of clinical pain, which triggers in two phases from the sc administration of formalin. The first (neurogenic) phase, which begins immediately after formalin administration, is characterized by direct activation of peripheral nociceptive fibers, especially C fibers. This neuropathic nociception inhibited by drugs capable of modulating neural pain mechanisms, such as opioids, that also control inflammatory nociception. The second (inflammatory) phase begins after 15 min of noxious stimulation, involving the activation of inflammatory pathways related to prostaglandins, serotonin, histamine, and NO. In this case, anti-inflammatory drugs can effectively control the nociception (Hunskaar and Hole, 1987; De Oliveira et al., 2015; Islam et al., 2019). AEVm (146.89 mg/kg) significantly (p < 0.001) reduces the nociception in FT second phase. However, this effect was not observed in the first phase. Similarly, in HP test, a thermogenic pain model that evokes supraspinatus nociception, therefore modulated by centrally acting analgesics, the extract did not increase latency for nociceptive reflex, i.e., paw lick or jump (Le Bars et al., 2001). In addition, opioid antagonist naloxone reversed the standard drug (morphine) effect in FT but did not modify the AEVm activity. These results allow us to deduce that the antinociceptive activity of AEVm involves peripheral mechanisms, especially inflammatory ones. It also reveals that the extract does not interfere with neurogenic nociceptive mechanisms and that opioid system is not involved in its effects.

Ultimately, to rule out the possibility of these effects being a product of locomotor capacity inhibition, we evaluated the ambulation of mice in open field for 5 min. The animals treated with the extract (146.89 mg/kg) presented locomotion, i. e., the total distance traveled, equivalent to the control group, allowing to conclude that it did not interfere with the locomotor behavior during nociception experiments. This finding is reinforced by the normality of tactile sensitivity and reflexes and absence of lethargy observed in the toxicity assessment, where animals were treated with 2,000 mg/kg of AEVm.

The anti-inflammatory activity of AEVm was evaluated in carrageenan-induced and dextran-induced paw edema and carrageenan-induced peritonitis in rats. Initial moments after phlogistic stimulation by intraplantar injection of carrageenan (0–2 h) are marked by the rise of inflammatory mediators such as histamine, serotonin and bradykinin, with increasing in local blood flow and capillary permeability, resulting in edema initiation (Sengar et al., 2015). In advanced stage (2–6 h) of inflammation, prostaglandin production, due to cyclooxygenase-2 (COX2) induction, and leukocyte migration are considered the key elements for its maintenance (Marzouk et al., 2010; Paiva et al., 2013). Our results revealed that AEVm (146.89 mg/kg) significantly inhibits edema formation in both stages with indomethacin-like effectiveness. Its efficacy in early stage is suggestive of histamine or bradykinin pathways inhibition, as well as possible stabilization of mast cells (Shashidhara et al., 2008). The persistence of the effect on late stage (2–5 h), with an average inhibition of about 74%, however, suggests possible interference with COX induction or activity. In dextran-induced model, whose edema formation occurs by induction of mast cell degranulation and release of histamine and serotonin (Ankier and Neat, 1972; Van Wauwe and Goossens, 1989), the involvement of these pathways in antiedematogenic effect of AEVm was evaluated. Our results demonstrated that the extract does not interfere with dextran-induced inflammation, indicating that histaminergic pathway is not involved in the antiedematogenic effect observed in the carrageenan-induced paw edema.

In the carrageenan-induced peritonitis test AEVm promoted potent inhibition of leukocyte migration and NO production. This test is an acute inflammation model, marked by intense leukocyte migration to the peritoneal cavity, induction of NO synthesis by induced nitric oxide synthase (iNOS) and COX2 expression, resulting in increased prostaglandin concentration. Indeed, several studies have shown the strong influence of NO production in prostaglandin levels, so that inhibition of NO synthesis reduces COX2 expression and prostaglandin production (Aquino et al., 2017). The ability of the extract to inhibit NO overproduction partly clarifies its mechanism of action and reinforces the hypothesis of the involvement of prostaglandin synthesis modulation in the antinociceptive and anti-inflammatory activities of AEVm.

In summary, our results demonstrate that the AEVm is rich in flavonoids, identified by UPLC-ESI-HRMS analysis, which potentially form the basis of its biological properties. They also demonstrated the safety of AEVm, that presented low oral acute toxicity, with antioxidant and hepatoprotective potential. Additionally, the extract has important peripheral antinociceptive and anti-inflammatory activity, which seems to be related to its constituent’s ability to inhibit NO and prostaglandin production, as well as inhibit leukocyte migration. Thus, the extract has shown promise for the development of new natural product aimed at treating pain and inflammation.

## Data Availability Statement

The datasets generated for this study are available on request to the corresponding author.

## Ethics Statement

The animal study was reviewed and approved by Ethic Committee on Animal Use of the Federal University of Para (CEUA/UFPA).

## Author Contributions

The study was conceived and designed by EF-J, CS, and MS. Experiments and data collection were performed by KL, FS-J, JO, TS, DA, SA, and PG. Data were analyzed by EF-J, KL, WP, MC, CM, CS, PG and MS. Drafting of the manuscript: KL and EF-J. Critical revision of the manuscript: EF-J and MS. All authors revised and approved the final version of the manuscript.

## Conflict of Interest

The authors declare that the research was conducted in the absence of any commercial or financial relationships that could be construed as a potential conflict of interest.
